# Successful neurolytic thoracic sympathetic ganglion block using C-arm fluoroscopic cone-beam computed tomography in patients with postmastectomy pain syndrome: a report of 3 cases

**DOI:** 10.1186/s40981-023-00639-3

**Published:** 2023-08-02

**Authors:** Shintaro Hagihara, Yoichiro Abe, Kohei Godai, Kyo Enohata, Akira Matsunaga

**Affiliations:** 1 Department of Anesthesiology and Pain Medicine, Kagoshima University Hospital, 8-35-1 Sakuragaoka, Kagoshima, 890-8520 Japan; 2 Department of Pain Clinic, NTT Medical Center Tokyo, 5-9-22 Higashigotanda, Shinagawa, Tokyo, 141-8625 Japan

**Keywords:** Postmastectomy pain syndrome, Thoracic sympathetic ganglion block, C-arm fluoroscopic cone-beam computed tomography, Sympathetically maintained neuropathic pain, Ethanol neurolysis

## Abstract

**Background:**

Postmastectomy pain syndrome involves persistent neuropathic and sympathetically maintained neuropathic pain that can be improved using a thoracic sympathetic ganglion block. However, conventional fluoroscopic procedures pose technical difficulties and are associated with potential severe complications. We report the use of C-arm fluoroscopic cone-beam computed tomography to enhance procedural success and treatment safety.

**Case presentation:**

Three women diagnosed with postmastectomy pain syndrome and experiencing persistent pain underwent C-arm fluoroscopic cone-beam computed tomography-assisted ethanol neurolytic thoracic sympathetic ganglion block. Pain severity decreased substantially after the procedure. The therapeutic effects were sustained for 12 months in cases 1 and 2 and for 5 months in case 3. All patients experienced a remarkable decrease in allodynia and hyperalgesia intensities.

**Conclusion:**

C-arm fluoroscopic cone-beam computed tomography-assisted neurolytic thoracic sympathetic ganglion block offers a valuable alternative for managing otherwise intractable postmastectomy pain syndrome before considering more invasive techniques.

## Background

Postmastectomy pain syndrome is one of the most intractable complications following breast cancer surgery [1, 2], defined as persistent pain after mastectomy/lumpectomy affecting the anterior thorax, axilla, and upper arm [2, 3]. Its etiology primarily involves neuropathic pain, and it is associated with sympathetically maintained neuropathic pain [2–12]. Although research on the treatment of postmastectomy pain syndrome is ongoing, no consensus has been reached [4–10]. Thoracic sympathetic ganglion block can offer an effective sympathetic block to improve sympathetically maintained neuropathic pain of postmastectomy pain syndrome by reducing the sympathetic outflow to the anterior chest wall [9–19]. However, conventional fluoroscopic procedures pose technical difficulties and can cause severe complications in anatomically difficult thoracic regions, such as the pneumothorax, neuraxial injection, intravascular injection, nerve laceration, neuritis, and Horner syndrome [15, 17, 20].

Alternatively, C-arm fluoroscopic cone-beam computed tomography (CBCT) with high-quality three-dimensional imaging technology has gained popularity in oncological and vertebral procedures, enabling precise monitoring of the needle location [21, 22]. Accurate needle placement is crucial for effective treatment; therefore, we speculate that CBCT image guidance can significantly contribute to the success and safety of thoracic sympathetic ganglion block. To our knowledge, this is the first report that involves CBCT-assisted single-injection ethanol neurolytic thoracic sympathetic ganglion block, which offers a valuable alternative for managing otherwise intractable postmastectomy pain syndrome before considering more invasive techniques.

## Case presentation

Written informed consent was obtained from the patients for the publication of this case report and the accompanying images. Three women diagnosed with postmastectomy pain syndrome were referred for the intervention. Patients’ general conditions are documented in Table 1. They presented with chronic neuropathic pain with allodynia and hyperalgesia in the precordial mastectomy and axillary lymphadenectomy regions, with scores ranging from 7 to 8 on the numerical rating scale (NRS) and were unresponsive to pharmacological and physical therapies. Second to fourth thoracic intercostal nerve blocks with 10 ml of 0.75% ropivacaine decreased the NRS scores to 4–5 but for a duration of only approximately 12 h. We planned to perform a single-injection ethanol neurolytic thoracic sympathetic ganglion block of the fourth thoracic sympathetic vertebra under CBCT guidance.

**Table 1:** General conditions and clinical courses of the patients

Case	1	2	3
Age (years)	44	44	50
Height (cm)	152	157	148
Weight (kg)	49	54	40
Surgical site	Left	Left	Right
Chemotherapy	-	+	+
Radiotherapy	-	-	+
Endocrine therapy	+	-	-
Duration of postmastectomy pain syndrome	14 months	4 months	25 months
Past medical history	Congenital deafness	Total hysterectomy	Hypothyroidism
Before thoracic sympathetic ganglion block			
NRS	8	8	7
Hyperalgesia	+	+	+
Allodynia	+	+	+
Medications	MGB 5 mg/day	PRG 150 mg/day	MGB 10 mg/day
		Tramadol 37.5 mg/day	DXT 20 mg/day
		APAP 325 mg/day	
		AMT 10 mg/day	
Thoracic sympathetic ganglion block			
Radiation dose	40.7 Gy.cm^2^	24.7 Gy.cm^2^	30.3 Gy.cm^2^
Fluoroscopy time	4 min 8 s	7 min 4 s	12 min 42 s
1 day after thoracic sympathetic ganglion block			
NRS	2	1	3
Hyperalgesia	-	-	-
Allodynia	-	-	-
Medications	MGB 5 mg/day	PRG 150 mg/day	MGB 10 mg/day
		Tramadol 37.5 mg/day	DXT 20 mg/day
		APAP 325 mg/day	
		AMT 10 mg/day	
1 month after thoracic sympathetic ganglion block			
NRS	2	1	3
Hyperalgesia	-	-	-
Allodynia	-	-	-
Medications	MGB 2.5 mg/day	Tramadol 37.5 mg/day	MGB 10 mg/day
		APAP 325 mg/day	DXT 20 mg/day
2 months after thoracic sympathetic ganglion block			
NRS	2	1	3
Hyperalgesia	-	-	-
Allodynia	-	-	-
Medications	MGB 2.5 mg/day	-	MGB 10 mg/day
			DXT 20 mg/day
6 months after thoracic sympathetic ganglion block			
NRS	2	1	4
Hyperalgesia	-	-	-
Allodynia	-	-	-
Medications	MGB 2.5 mg/day	-	MGB 10 mg/day
			DXT 20 mg/day
12 months after thoracic sympathetic ganglion block			
NRS	2	1	4
Hyperalgesia	-	-	-
Allodynia	-	-	-
Medications	MGB 2.5 mg/day	-	MGB 10 mg/day
			DXT 20 mg/day

*NRS*, numerical rating scale; *MGB*, mirogabalin; *PRG*, pregabalin; *DXT*, duloxetine; *APAP*, acetaminophen; *AMT*, amitriptyline

**Fig. 1:**
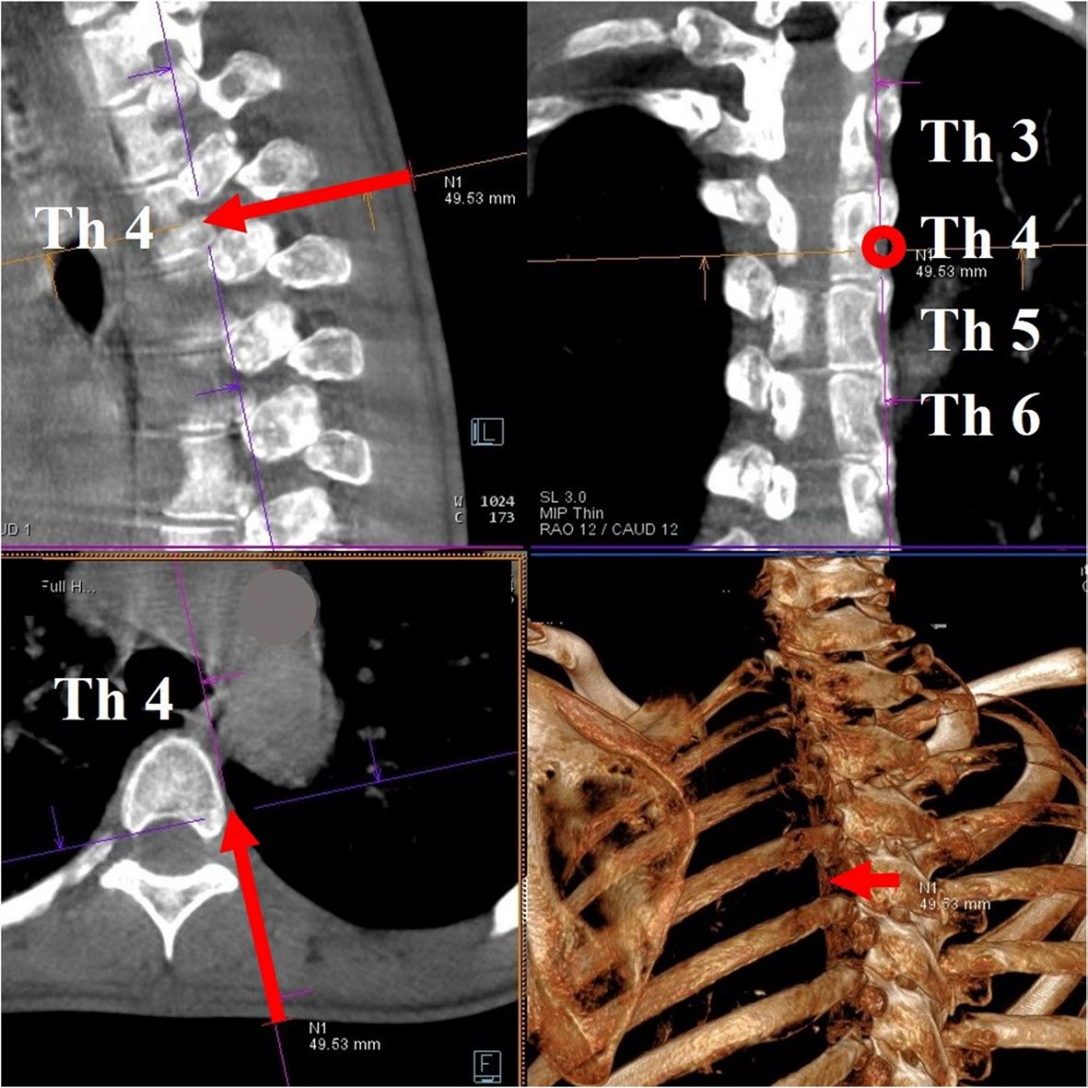
The planned needle path. The arrow and circle show the planned needle path for the posterolateral approach at the fourth thoracic vertebra in sagittal, coronal, and axial planes and three-dimensional multiplanar reconstructed images

**Fig. 2:**
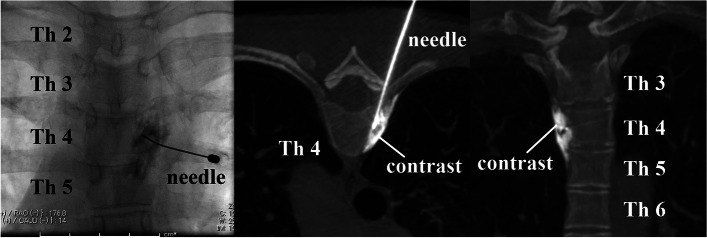
Needle deployment and the distribution of contrast medium. Contrast medium spreads between the thoracic vertebra and radiates sternocostal ligament at the fourth thoracic vertebra in fluoroscopy and axial and coronal multiplanar reconstructed images

Procedures were performed under light sedation with an intramuscular injection of hydroxyzine 25 mg. The patients were placed in the prone position. Two board-certified interventional radiological technologists performed the rotational acquisition of CBCT images of the upper thoracic spine using Artis Zeego (Siemens Healthineers, Erlangen, Germany). Multiplanar reconstructed images were obtained to plan the desired skin entry and target points for the posterolateral approach (Fig. 1). The C-arm was positioned where the entry and target points directly superimposed along the planned needle path. A small circle marked on the fluoroscopic image was used to indicate the target point. For skin and soft tissue anesthesia, 1% mepivacaine was administered. A 22-gauge guiding needle (Hakko Medical, Nagano, Japan) was advanced according to the planned needle path. A second CBCT scan was performed to revise for the positional correlation between the actual needle tip and the desired target point. The needle was advanced for minor adjustments, and 1 ml of the contrast medium along with 2% mepivacaine was injected under live fluoroscopy to ensure no flow into the intravascular space. A third CBCT scan was performed to confirm that the contrast medium did not spread into the intrapleural, epidural, and intercostal spaces (Fig. 2). After confirming the absence of procedure-related complications, 0.5 ml of 95% ethanol was injected. The patients were placed in bed to rest for 2 h, and anesthesia recovery was observed. Spinal cord and intercostal nerve neuritis symptoms were not observed.

**Fig. 3:**
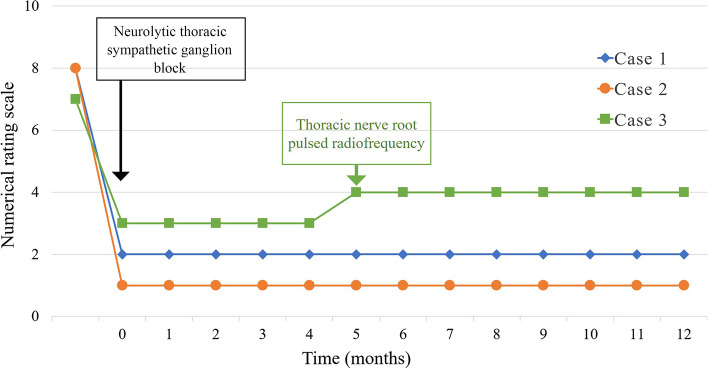
Clinical course of each patient. Line graphs show the change of numerical rating scale (NRS) pain scores. In cases 1 and 2, the NRS scores remained stable after  neurolytic thoracic sympathetic ganglion block. However, in case 3, the NRS score increased to 4 after 5 months, and a third thoracic nerve root pulsed radiofrequency was performed; thereafter, the same pain scores remained stable

On the day after the procedure, the NRS scores in cases 1–3 decreased from 8 to 2, 8 to 1, and 7 to 3, with a remarkable decrease in allodynia and hyperalgesia intensity in all patients. In cases 1 and 2, the NRS scores remained stable, and analgesic consumption decreased over the course of the next 12 months. However, in case 3, the NRS score increased to 4 after 5 months. We performed a third thoracic nerve root pulsed radiofrequency using 3 ml of 1% mepivacaine; thereafter, the same pain scores remained stable. Table 1 and Fig. 3 describe the pain scores and clinical courses until the 12-month follow-up visit.

## Discussion

This case report presents two important findings. First, CBCT-assisted ethanol neurolysis of thoracic sympathetic ganglion block can alleviate intractable postmastectomy pain syndrome. Second, real-time CBCT image guidance can provide technical success with increased accuracy when placing the needle for thoracic sympathetic ganglion block.

Preferred first-line treatments for postmastectomy pain syndrome include pharmacological, physical, and multidisciplinary combination therapy [6, 7]. Less invasive options of peripheral nerve blocks, such as intercostal nerve block and serratus plane block, can alleviate neuropathic pain by suppressing afferent nociceptive signals and inflammatory reactions and prevent the integration of nociceptive impulses into the central nervous system [6–16]. For patients with refractory pain, more invasive techniques of thoracoscopic sympathectomy and spinal cord stimulation can be considered [9, 15]; however, intractable pain is an occasional outcome even after spinal cord stimulator implantation [17].

Sympathetic blocks can be used for the treatment of intractable postmastectomy pain syndrome [9–20]. However, conventional fluoroscopic or computed tomography (CT)-guided thoracic sympathetic ganglion blocks may cause severe complications [15, 17–23]. A study on conventional fluoroscopic neurolytic thoracic sympathetic ganglion block reported complications in 7.5% of the procedures, suggesting that the correct needle tip positioning and the right distribution of the contrast medium should be confirmed three-dimensionally before using neurolysis [20]. Another study on CT-guided thoracic sympathetic ganglion block reported adverse events in 7.1% of the procedures [15]. Alternatively, CBCT provides higher spatial resolution than CT and allows for precise evaluation of small and complex anatomical structures that cannot be detected using conventional fluoroscopy [21–24]. Needle guidance using CBCT has an accuracy of approximately 3 mm, whereas that using CT results in up to 7 mm of deviation from the target [24]. Radiation exposure during CBCT imaging is 13–42% lower than that during CT guidance [24, 25]; however, needle placement time does not differ [24]. In this report, the second CBCT can lead to pinpoint accuracy of the needle tip to reach the desired location, and the third CBCT can ensure the right distribution of the contrast medium three-dimensionally. The mean radiation dose and fluoroscopy time were 31.9 Gy.cm^2^ (range 24.7–40.7 Gy.cm^2^) and 7 min 58 s (4 min 8 s–12 min 42 s), respectively. A learning curve exists before CBCT guidance can be used efficiently [21]. We speculate that CBCT image guidance can significantly contribute to the success and safety of ethanol neurolytic thoracic sympathetic ganglion block. Further studies are required to confirm the procedure’s clinical relevance.

Neurolysis can be used for semipermanent methods of nerve damage [10–13], as it interrupts the pain signal transmission via Wallerian degeneration distal to the injection site [13]. Radiofrequency ablation of the second to fourth thoracic sympathetic ganglia and cryoneurolysis of the intercostobrachial nerve are reported to reduce pain in patients with postmastectomy pain syndrome [10, 11, 26]. However, chemical neurolysis in thoracic sympathetic ganglion blocks performed at the third or high upper thoracic vertebral level is unsafe, given that the resulting Horner syndrome can affect patients’ quality of life [14, 15]. One study reported that permanent Horner syndrome occurred in relation to the second thoracic ganglion [15]. Therefore, we administered ethanol neurolysis after the contrast medium spreads lower than the third thoracic vertebra, to ensure that the correct regions were targeted. We speculate that CBCT-assisted single-injection ethanol neurolytic thoracic sympathetic ganglion block of the fourth thoracic ganglion may be safe for patients with Horner syndrome. Further studies are required to confirm the optimal ethanol dose.

Thoracic sympathetic ganglion block alleviating allodynia and hyperalgesia intensity suggests that the sympathetic nervous system can be a therapeutic target for postmastectomy pain syndrome [11, 15]. The sympathetic nervous system depresses acute pain perception via descending inhibition of nociception [13, 27]. Paradoxically, chronic activation of the sympathetic nervous system may augment pain and lead to sympathetically maintained neuropathic pain [15, 28]. The underlying mechanism for this pain is unclear; however, it may be caused by abnormal coupling between the sympathetic and somatosensory nervous systems due to neurogenic inflammation [11, 28]. Local inflammation may activate the surrounding glia and induce sprouting of sympathetic nerve fibers, which may provide excitatory inputs to dorsal root ganglion neurons and further provoke the inflammation process [29]. These interactions may play an important role in the initiation of chronic pain, allodynia, and hyperalgesia [27–29]. CBCT-assisted ethanol neurolytic thoracic sympathetic ganglion block may be a useful alternative for intractable postmastectomy pain syndrome as it alleviates sympathetically maintained neuropathic pain.

In conclusion, CBCT-assisted ethanol neurolytic thoracic sympathetic ganglion block can offer a valuable alternative for otherwise intractable postmastectomy pain syndrome before considering more invasive techniques.

## Data Availability

Data sharing is not applicable to this article as no datasets were generated or analyzed during the current study.

## Abbreviations

CBCT: C-arm fluoroscopic cone-beam computed tomography
CT: Computed tomography
NRS: Numerical rating scale
